# *QuickStats:* Percentage* of Adults with Fair or Poor Health,^†^ by Home Ownership Status^§^ and Age Group — National Health Interview Survey, United States, 2019^¶^

**DOI:** 10.15585/mmwr.mm7013a7

**Published:** 2021-04-02

**Figure:**
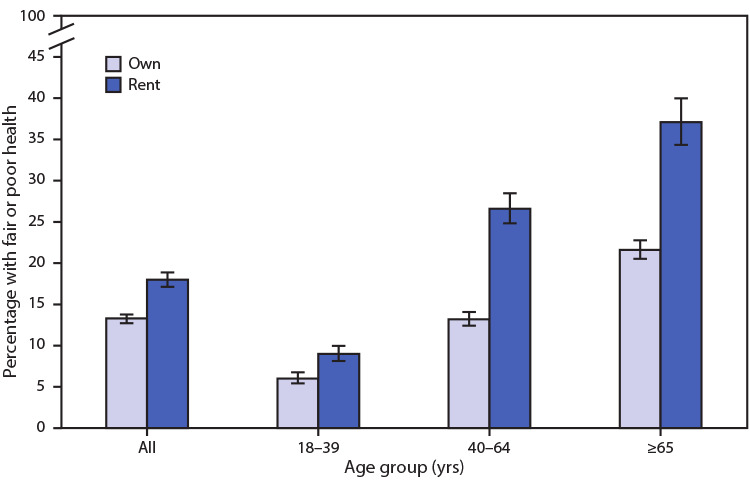
In 2019, 18.0% of renters assessed their health as fair or poor, compared with 13.3% of homeowners. For each age group, renters were more likely than homeowners to report fair or poor health: 9.0% versus 6.0% among adults aged 18–39 years, 26.6% versus 13.2% among those aged 40–64 years, and 37.1% versus 21.6% among those aged ≥65 years. For both renters and homeowners, the percentage of adults with fair or poor health increased with increasing age.

**Source:** National Health Interview Survey, 2019. https://www.cdc.gov/nchs/nhis.htm

